# Preoperative thickness and postoperative atrophy of the abdominal rectus muscle as risk factors for parastomal hernia after colostomy creation

**DOI:** 10.1007/s10029-025-03482-w

**Published:** 2025-10-10

**Authors:** Johan Nyman, Kristoffer Huss, Lennart Flygare, Karin Strigård

**Affiliations:** 1 Department of Diagnostics and Intervention, Umeå University, Umeå, 901 85 Sweden; 2 Sunderby Research Unit, Norrbotten County Council, Umeå University, Umeå, Sweden

**Keywords:** Parastomal hernia; colostomy, Abdominal rectus muscle, Rectal cancer

## Abstract

**Purpose:**

Parastomal hernia (PSH) is a common and often burdensome stoma complication. Surgical repair carries high morbidity and mortality. Hence, prevention of PSH formation would be ideal, which requires better understanding of risk factors. We aimed to examine the role of abdominal rectus muscle (ARM) thickness and its postoperative atrophy in PSH pathogenesis. We hypothesised that a thin ARM upon stoma creation is a risk factor for PSH development, and that patients developing PSH show a higher degree of postoperative ARM atrophy compared to patients without PSH.

**Methods:**

Radiological bilateral ARM measurements on 205 patients, before and after rectal cancer surgery with Hartmann’s procedure or abdominoperineal resection, among Swedish patients recruited retrospectively from the Swedish Colorectal Cancer Registry. Hypotheses were tested using *t*-test and multivariable logistic regression.

**Results:**

Patients developing PSH had a greater degree of ARM atrophy than hernia-free patients (-3.2 mm [-36.4%] vs. -1.6 mm [-18.2%]; *p* = 0.002). Postoperative ARM atrophy (OR 1.17; 95% CI 1.05–1.31, *p* = 0.006), body mass index (OR 1.15; 95% CI 1.06–1.24, *p* < 0.001) and laparoscopic approach (OR 2.60; 95% CI 1.27–5.31, *p* = 0.009) were independent risk factors for PSH in the multivariable model. Preoperative anteroposterior thickness of the ARM was not found to be a risk factor.

**Conclusion:**

Patients developing PSH had a more pronounced atrophy of the ipsilateral ARM than patients without PSH. A thinner ARM preoperatively did not increase the risk for PSH. Further research should focus on what causes postoperative ARM atrophy.

## Introduction

Parastomal hernia (PSH) affects approximately half of stoma patients within two years of stoma creation [1]. Not all patients with PSH have symptoms, but PSH patients generally experience impaired quality of life [2–5]. Symptoms include dressing difficulties causing dermatitis, odour, and social isolation, as well as cosmetic concerns, problems with the bulging itself, and in some cases even life-threatening incarceration [6].

Surgical repair of PSH is associated with relatively high morbidity and mortality, with a 13% 30-day reoperation rate and a 6% 30-day mortality rate [7]. Given these unsatisfactory figures, it would be ideal to find a way to prevent PSH development. This necessitates a good understanding of risk factors, which in the literature includes high body mass index (BMI), high age, female sex, larger aperture size and larger waist circumference [8]. Some aspects of surgical technique have been investigated; neither positioning of the stoma through the abdominal rectus muscle (ARM) compared to lateral to it [6], nor the method of rectus sheath opening [9] having shown any clear importance. It has been suggested that patients undergoing laparoscopic surgery might be at higher risk for PSH compared to patients undergoing open surgery [10, 11].

Stomas are routinely passed through the ARM that is arterially supplied by the deep inferior epigastric artery and innervated by spinal nerves, all of which travel along the dorsolateral aspect of the ARM within the rectus sheath [12]. Most nerve fibres innervate only small portions of the ARM, but some nerve branches innervate the full thickness of the ARM at that segment. This implies that nerve damage could result in total ARM denervation at that segment and caudally [13]. Studies suggest that colostomies tend to medialise over time [14] and that ARM atrophy might play a role in PSH pathogenesis although results are conflicting [15, 16]. It is not clear whether the properties of the ARM or operative trauma to it during stoma creation affects the risk for developing PSH.

We hypothesised that a thin ARM preoperatively is a risk factor for PSH development after stoma creation, and that there is greater atrophy of the ARM after stoma creation in patients who develop PSH compared to patients who do not.

## Methods

In this retrospective cohort study, the Swedish Colorectal Cancer Register (SCRCR) was searched for rectal cancer resections in Västerbotten County, Sweden, between the years 2004–2021. The SCRCR is a nationwide register where all Swedish colorectal cancer cases are recorded. The coverage was > 95% for the entire study period [17, 18]. The register holds clinical and demographic data such as tumour stage, pathology, radiology, oncological treatment, short- and long-term follow-up data.

Rectal cancer patients undergoing curative tumour resection with permanent end colostomy creation were included. The availability of abdominal computerised tomography (CT) images prior to surgery and 9–18 months postoperatively was mandatory for inclusion. Only patients with stomas placed through the ARM were included. Patients undergoing divertive stoma surgery prior to tumour resection or abdominal surgery such as reoperation or stoma revision between preoperative and postoperative CT examinations, were excluded.

## Radiological examination

There is no established gold standard for PSH diagnosis, CT being the main modality used in routine clinical practice to confirm a clinically suspected PSH [1]. Sensitivity is increased with CT compared to clinical examination alone [19], but one drawback is that a PSH sometimes cannot be distinguished from a peristomal bulging or stoma prolapse. The sensitivity and specificity of CT for PSH diagnosis have been estimated at 94% and 50%, respectively [20].

In Sweden, colorectal cancer patients are routinely scanned at one and three years postoperatively as part of the oncological follow-up. Pre- and postoperative images were collected from Umeå University Hospital, Lycksele Hospital, and Skellefteå Hospital; all in Västerbotten County, Sweden. If several preoperative CT examinations were available, the one closest to surgery was chosen. Postoperative examinations were the routine oncological one-year follow-up scans. To allow flexibility, we decided to include patients undergoing postoperative CT at 9–18 months postoperatively. Scans with and without intravenous contrast medium were included. Since scans were for oncological purposes, hernia examination in the prone position or with Valsalva manoeuvre was not done. All cases were radiologically reviewed by author KH. Equivocal cases were reviewed collectively by authors JN, KH, and KS to reach consensus.

**Fig. 1:**
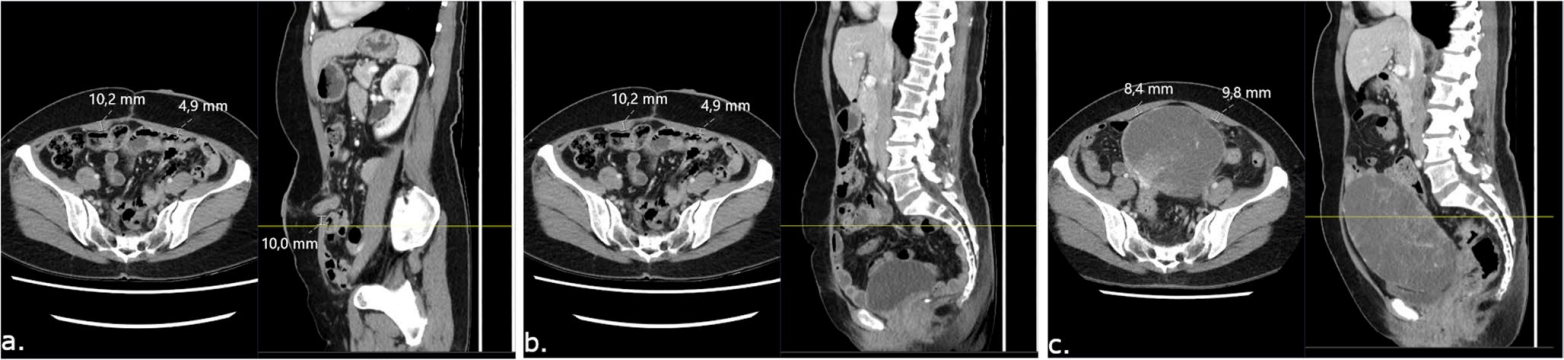
Abdominal rectus muscle (ARM) measurements on computerised tomography (CT) scans of rectal cancer patients subjected to tumour resection with permanent end colostomy. First, on postoperative CT **(a.)**, the location of the stoma is identified, and ARM measurements are made on axial slices 1 cm caudal to the stoma aperture. On sagittal slices **(b.) **the craniocaudal level at which the measurements were made is then established in relation to fix structures such as the vertebral column. Then, on preoperative imaging **(c.) **the same craniocaudal level is identified and ARM measurements are made on axial slices the same way as in **(a.)**

First, postoperative images with the stoma in situ were reviewed to establish the cranio-caudal location of the stoma within the ARM, using the spinal vertebrae as reference. The presence of PSH was evaluated, defining PSH as any herniation of intra-abdominal contents ventral to the anterior rectus sheath. Stoma prolapse as well as the inevitable presence of stoma intestinal mesentery was not considered a PSH. On axial slices, anteroposterior (AP) thickness of the ARM was measured using a picture archive and communications system (PACS) [SECTRA, Linköping, Sweden]. The AP thickness of the ARM (ARM-AP) was defined as the muscle including the rectus sheath. Measurements were made on axial slices one centimetre caudal to the caudal-most aspect of the stoma orifice, at the latero-lateral point where the ARM was thickest. The rationale behind measuring one centimetre caudal to the stoma orifice was that neuroanatomically, atrophy of the ARM should occur at the level of neurovascular compromise and caudal to that. Furthermore, the region immediately adjacent to the stoma orifice would be affected by fibrosis and some degree of distortion, making measurements difficult to interpret. The same ARM measurements were made on preoperative scans. To ensure that measurements were made at the same cranio-caudal and latero-lateral levels as with postoperative images, the vertebral column and surrounding structures were used as references to identify the corresponding axial slices. Measurements of the contralateral ARM were made at the same cranio-caudal level, measuring the AP thickness at the thickest portion of the muscle at this level. The ARM measurement procedure is illustrated in Fig. 1. Differences between preoperative and postoperative ARM-AP (ΔARM-AP [mm]) measurements were calculated bilaterally in all cases.

Cases of PSH were classified according to the EHS classification of parastomal hernias [21].

## Statistics

Data were collected in a Microsoft Excel (Microsoft Corp., Redmond, WA, USA) database and statistical analyses conducted in IBM SPSS 28 (IBM Corp., Armonk, NY, USA). Hypotheses were tested using logistic regression and *t*-test. The known potential risk factors age, sex, BMI and laparoscopic approach were included in analyses. Variables were first tested in a univariable model, then in a multivariable model using the enter method. Since ARM-AP and ΔARM-AP were not independent of each other, they were analysed in separate multivariable models. Categorical and continuous variables were tested using the *Χ*^*2*^ and *t*-test, respectively. In cases of missing data, the case was excluded from analysis of that particular variable. There were no missing data relating to the primary or secondary hypotheses.

To evaluate the intra-observer reliability of radiological measurements, repeat measurements of preoperative ipsilateral ARM-AP were made six months after index measurements for 20 (approximately 10% of cohort) randomised patients. The intraclass correlation coefficient (ICC) was calculated.

A formal power calculation was not feasible since information on association between ARM thickness, atrophy and PSH is scarce. Instead, we pragmatically chose a sample size of 200 cases since no difference in a sample of 200 patients would suggest no clinical significance in this context.

## Compliance with Ethical Standards

The study was approved by the Swedish Ethical Review Authority (diary number 2020-05057), waiving the need for informed consent due to retrospective design; and conducted and reported in accordance with the Declaration of Helsinki [22] and the STROBE statement [23].

## Results

A total of 205 cases were included, of which 80 (39%) were female. Surgical procedures undertaken were either abdominoperineal rectal resection or Hartmann’s procedure (181 and 24 cases, respectively). The two groups, PSH and non-PSH, were demographically and clinically similar except for mean BMI being significantly higher in the PSH group (27.8 [SD 5.1] vs. 25.6 [SD 4.1] in the non-PSH group; p = 0.006), and that laparoscopic surgery was more common in the PSH group (48% vs. 26%, p = 0.005). The mean age for the entire cohort was 68 years (SD 9.9). Fifty patients (24%) had developed PSH at the time of postoperative oncological follow-up CT, of which seven (14%) had a concomitant incisional hernia after open surgery. Table 1 shows further demographic and clinical details.

**Table 1:** Demographic and clinical data

		PSH (N = 50)	No PSH (N = 155)	*p*
Sex	**Male**	27 (54)	98 (63)	0.245
	**Female**	23 (46)	57 (37)	
Age		67.9 (10.2)	67.9 (9.9)	0.969
BMI		27.8 (5.1)	25.6 (4.1)	**0.006**
Procedure	**APR (N = 181)**	48 (96)	133 (86)	0.051
	**Hartmann (N = 24)**	2 (4)	22 (14)	
pTNM	**T0**	4 (8)	4 (3)	0.339
	**T1**	3 (6)	9 (6)	
	**T2**	10 (20)	38 (25)	
	**T3**	27 (54)	87 (56)	
	**T4**	2 (4)	13 (8)	
	**TX**	2 (4)	2 (1)	
	**N0**	31 (62)	90 (58)	0.099
	**N1**	13 (26)	30 (19)	
	**N2**	4 (8)	33 (21)	
	**M0**	41 (82)	139 (91)	0.487
	**M1**	7 (14)	14 (9)	
Neoadjuvant therapy	**Chemotherapy**	5 (10)	30 (19)	0.122
	**Radiotherapy**	38 (76)	121 (78)	0.646
Laparoscopy including robot-assisted		24 (48)	41 (26)	**0.005**
Surgical complications within 30 days		17 (34)	40 (26)	0.261
EHS classification	**I**	30 (60)	N/A	N/A
	**II**	6 (12)		
	**III**	13 (26)		
	**IV**	1 (2)		
Concomitant incisional hernia		7 (14)	N/A	N/A

Categorical variables are expressed as N (%). Continuous variables are expressed as mean (SD). Statistical tests used are *Χ*^2^ and independent samples *t*-test, as appropriate

*PSH *parastomal hernia; *APR *abdominoperineal rectal resection; *BMI *body mass index (kg/m^2^); *pTNM *pathologic tumour, node and metastasis stage; *EHS *European Hernia Society

Patients underwent preoperative CT at a median of 28 days (range 0–403, IQR 19–46) preoperatively. The wide range is explained by three outliers for whom no new CT was made preoperatively but only pelvic magnetic resonance imaging (MRI). Postoperative CT was performed at a mean of 12 (range 9–17) months postoperatively.

In three cases it was initially difficult to judge whether a PSH was present. Those cases were eventually decided through unanimous consensus between authors KH, JN and KS.

The group of patients that developed PSH had a greater postoperative reduction (i.e. atrophy) in ipsilateral ARM thickness than hernia-free patients; −3.2 mm vs. −1.6 mm (−36.4% vs. 18.2%), respectively (p < 0.001) [Table 2]. On the contralateral side, there was no atrophy seen at group level, but rather a slight increase in thickness with no statistically significant difference between groups; +0.4 mm vs. +0.5 mm for the PSH group and non-PSH group respectively (p = 0.70).

**Table 2:** Cange in anteroposterior abdominal rectus muscle thickness (ARM-AP) between preoperative and postoperative CT images, according to parastomal hernia (PSH) development

**Ipsilateral side ARM-AP (mm)**						
		Preoperative	Postoperative	Mean difference (mm)	Mean difference (%)	*p*
PSH	**Yes**	8.8	5.6	−3.2	−36.4	**< 0.001**
	**No**	8.8	7.2	−1.6	−18.2	
**Contralateral side ARM-AP (mm)**						
		**Preoperative**	**Postoperative**	**Mean difference (mm)**	**Mean difference (%)**	***p***
PSH	**Yes**	10.2	10.6	+ 0.4	+ 3.9	0.70
	**No**	9.8	10.3	+ 0.5	+ 5.1	

Numbers are means and *p*−values are derived from independent samples *t*−tests

In the multivariable logistic regression model, postoperative atrophy of the ipsilateral ARM was found to be an independent predictor of PSH development (OR 1.17; 95% CI 1.05–1.31, p = 0.006). Higher BMI was also an independent predictor (OR 1.15; 95% CI 1.06–1.24, p < 0.001), while age and female sex were not. The risk for PSH was significantly higher for stomas placed as part of a laparoscopic procedure, compared to open surgery (OR 2.60; 95% CI 1.27–5.31, p = 0.009). Preoperative ARM-AP was not found to be an independent risk factor for PSH (OR 0.98; 95% CI 0.87–1.11, p = 0.78) [Table 3].

**Table 3:** Univariable and multivariable logistic regression analysis of potential predictors of parastomal hernia

	Univariable model		Multivariable models†			
	OR (95% CI)	*p*	Model A		Model B	
			OR (95% CI)	*p*	OR (95% CI)	*p*
Age	1.00 (0.97–1.03)	0.97	1.00 (0.97–1.04)	0.85	1.01 (0.98–1–05)	0.47
Female	1.47 (0.77–2.79)	0.25	1.76 (0.80–3.87)	0.16	2.00 (0.97–4.11)	0.06
BMI	1.12 (1.04–1.20)	**0.004**	1.15 (1.06–1.25)	**< 0.001**	1.15 (1.06–1.24)	**< 0.001**
Laparoscopy	2.52 (1.30–4.88)	**0.006**	2.96 (1.45–5.94)	**0.002**	2.60 (1.27–5.31)	**0.009**
ARM-AP	1.01 (0.91–1.11)	0.91	0.98 (0.87–1.11)	0.78	-	-
ΔARM-AP	1.18 (1.07–1.31)	**0.001**	-	-	1.17 (1.05–1.31)	**0.006**

† Multivariable analyses were made separately for two models, including the variables ARM−AP (Model A), and ΔARM−AP (Model B), respectively

*ΔARM−AP* ipsilateral change in anteroposterior thickness of the abdominal rectus muscle between preoperative and postoperative scans (mm)

*ARM−AP* ipsilateral preoperative anteroposterior thickness of the abdominal rectus muscle (mm)

*BMI* body mass index (kg/m2)

When repeat measurements of the preoperative ipsilateral ARM-AP were made six months after index measuring, the resulting ICC value was 0.97 (95% CI 0.92–0.99, p < 0.001).

## Discussion

In this study, patients who developed PSH after rectal cancer resection with permanent colostomy showed greater postoperative ARM atrophy than patients not developing PSH, and postoperative ARM atrophy was an independent predictor of PSH. Preoperative ARM thickness was not found to be useful in predicting PSH development.

These findings indicate that ARM atrophy might play a role in PSH pathogenesis, highlighting the need for targeted studies investigating this association and potentially causal relationship.

There may be several possible reasons for atrophy in this context, one of which being neurovascular damage at stoma creation. Rozen et al. [13] showed the risk of complete ARM denervation if a larger nerve in the infra-umbilical ARM, usually located at the level of the arcuate line, is damaged. Injudicious opening of the rectus sheath could result in cauterisation or damage to the deep inferior epigastric artery or its branches as well as closely associated nerves, resulting in compromised blood flow to the ARM and/or direct nerve damage. Similarly, excessively forceful stomal aperture dilation could cause shear trauma to the above-mentioned structures. Many of the known risk factors behind PSH are non-modifiable, but the abovementioned technical factors are all under the control of the operating surgeon.

There are other factors that could affect the risk for muscle atrophy in general, including malnutrition and systemic medication such as corticosteroids and chemotherapy. Some degree of postoperative decrease in ARM-AP was seen on the ipsilateral side in both the PSH and non-PSH groups, but none was seen on the contralateral side. This suggests that atrophy on the ipsilateral side was not caused by systemic factors such as chemotherapy, malnutrition or postoperative sarcopenia, since these would affect both sides. Nagayoshi et al. [15] found that patients with PSH, on the group level, had a thinner ARM caudal and medial to the stoma postoperatively as compared to patients without PSH. That study included 91 cases and could only demonstrate atrophy on the medial caudal aspect of the stoma, with a borderline significant p value, but not on the lateral side. Measurements were made postoperatively only, making it difficult to categorically say that there was an actual decrease in muscle thickness over time. In a study on prophylactic retromuscular mesh at stoma creation, Täckström et al. [16] found mesh placement to be associated with ARM atrophy, which in turn surprisingly was protective against PSH. In that study, subjective visual assessments, rather than measurements, were made to judge atrophy. Timmermans et al. [14]. examined ARM atrophy in relation to PSH but could not demonstrate any postoperative atrophy on the side ipsilateral to the stoma in the PSH group, maybe due to lack of power (n = 30).

Another possible explanation could be that a PSH itself compromises the function of the ARM, and even in the absence of a hernia, the very presence of a stoma might impair effective activation of the ARM. To our knowledge no study has examined this aspect functionally. The fact that a slight increase in contralateral ARM thickness was seen might lend support to this idea. Should it be that dysfunctional activation of the ARM is a contributing factor to postoperative atrophy, then theoretically this could be avoided by postoperative physiotherapy.

A large hernia might distort the abdominal wall visually, affecting radiological measurements. However, since the rectus sheath is a closed room, it seems unlikely that a displaced ARM would appear thinner. Rather, the ARM would be compressed and appear thicker. To minimise any distortion effect on CT evaluations, measurements were made one centimetre caudal to the caudal-most aspect of the stoma aperture.

Preoperative ARM thickness did not predict PSH. Neither did female sex, contrary to previous reports [8]. A high BMI was found to be a risk factor, as frequently reported in the literature. Laparoscopic approach was also an independent predictor of PSH, consistent with some previous reports [10, 11, 24]. The mechanism behind the increased risk of PSH among laparoscopically created stomas is not known. Shiraishi et al. [24]. found that PSH is more likely to develop if a stoma does not pass through the mid-ARM. Since accurate placement of the stoma can sometimes be difficult with a laparoscopic approach, this might result in the stoma not passing through the mid-ARM. In that study, the mid-ARM was defined as the mid third of the latero-lateral width of the ARM as measured on axial CT slices. With intra-abdominal gas insufflation during laparoscopy, the abdominal wall is significantly distended which might mislead the surgeon when dissecting the subcutaneous tissue and subsequently choosing the incision site in the rectus sheath, especially in obese patients. Theoretically, a stoma passing through the lateral third of the ARM would increase the risk for neurovascular damage, thus supporting the neurovascular theory, but data regarding the distribution of stoma placement was not provided in that report. Another theory is that laparoscopic stoma creation can result in the aperture to be too large, also due to the abdominal distension caused by gas insufflation. Additionally, minimally invasive surgery significantly reduces adhesion formation, which may result in the stomal bowel and peristomal tissue becoming less fixed and, consequently, more susceptible to weakening and herniation.

This study is limited by its retrospective design, primarily since CT examinations were not conducted for the purpose of this study, hernia protocols not being used in any of the cases. This implies a risk of non-differential misclassification bias as the PSH incidence in our material might be slightly underestimated. The statistical implication of this would be a potential attenuation of the associations between PSH and the identified risk factors, rather than false associations. Another limitation related to retrospective design is non-standardisation of the stoma creation technique. Thus, different surgeons and institutions may in theory have created stomas somewhat differently. We have not, however, seen any proof of any differences in outcomes between different hospitals. The period of this study was almost two decades, and CT scanners obviously improve over time. The main difference between older and newer scanners lies in image post-processing. For some older scanners in this study, slice thickness was slightly thicker than what is routine nowadays. We argue, however, that the technical differences were of insignificant importance since the abdominal wall was easily and clearly visualised in all cases, even in the absence of contrast medium. As with any kind of measurement and imaging interpretation, some degree of subjectivity was inevitable. The same author (KH) evaluated all images and made measurements, reaching an almost perfect intra-observer reliability with an ICC value of 0.97 when repeating measurements six months after index measurements. This strongly suggests that measurements were sufficiently standardised and that precise, detailed and reproducible measuring was feasible in the PACS.

Imaging was evaluated at one year after surgery, and 24% of patients were found to have a PSH, somewhat lower than the 30% estimated in the literature [1]. This might be due to CT scanning in this study not being aimed at hernia diagnosis. Furthermore, our cohort comprised patients operated on with curative intent, a generally healthier group probably at less risk for PSH formation. The rationale for choosing one year postoperatively for measurements was that PSHs usually develop within the first two postoperative years [25]. A longer observation period would increase the risk of introducing confounding factors that could also affect hernia development or muscular atrophy. Furthermore, at three years postoperatively some patients would have undergone PSH repair or other significant abdominal surgery. It also seemed reasonable to let the operative area recover before making radiological measurements. We thus considered one year postoperatively to be the point in time when the region of interest had recovered while avoiding any confounding factors.

Regarding the time frame for preoperative imaging, there were three outliers for whom the interval between preoperative CT imaging and surgery was significantly longer than for the rest of the cohort. The reason for this was that no repeat CT was made prior to surgery for these patients, but only MRI, which occasionally was the case in that period of time prior to standardisation of rectal cancer management in Sweden. When analysing this outlier group descriptively it did not differ from the rest of the cohort and when conducting sensitivity analyses with the outliers excluded, results were not altered in any way. We thus chose to keep these cases in analyses.

An important strength of this study is that all cases were included from a continuously validated national register with almost 100% coverage, meaning that clinical and demographic data are trustworthy and that the risk of significant selection bias is minimal. Significantly more cases were included in this study, compared to the abovementioned previously published studies on ARM thickness and atrophy in relation to PSH. Also, measurements were made on both preoperative and postoperative images, enabling appropriate statistical analyses of atrophy as a change in thickness over time. Furthermore, radiological analyses were standardised and performed by the same researcher to minimise intra-observer variation, as reported above. The cohort of patients studied was homogenous with respect to surgical procedures and oncological treatments. Since calculation of sample size and power was deemed too arbitrary and thus not undertaken, there was a risk for type 2 error. We believe, however, that a risk factor not showing significance in over 200 cases has limited use in everyday clinical practice.

## Conclusions

Postoperative atrophy of the ARM after colostomy creation is associated with PSH development. Preoperative AP thickness of the ARM is not a predictor of PSH development. Further research should focus on determining the cause of ARM atrophy after stoma creation and how it can be prevented, diagnosed, and treated; as well as investigate the natural course of abdominal wall changes after stoma creation.

## Data Availability

Provision of anonymised data will be considered upon reasonable request.

## Declarations

### Statements and declarations

The authors have no conflicting interests.
